# Adoptive Cell Therapy in Breast Cancer: A Current Perspective of Next-Generation Medicine

**DOI:** 10.3389/fonc.2020.605633

**Published:** 2020-10-27

**Authors:** Jesús Fuentes-Antrás, Kissy Guevara-Hoyer, Mariona Baliu-Piqué, José Ángel García-Sáenz, Pedro Pérez-Segura, Atanasio Pandiella, Alberto Ocaña

**Affiliations:** ^1^ Breast Cancer Unit, Medical Oncology Department, San Carlos University Hospital, Madrid, Spain; ^2^ Experimental Therapeutics and Translational Oncology Unit, Medical Oncology Department, San Carlos University Hospital, Madrid, Spain; ^3^ Clinical Immunology Department, San Carlos University Hospital, Madrid, Spain; ^4^ Institute of Molecular and Cellular Biology of Cancer and Centro de Investigación Biomédica en Red de Cáncer (CIBERONC), Consejo Superior de Investigaciones Científicas (CSIC), Salamanca, Spain

**Keywords:** adoptive cell therapy, breast cancer, TIL, TCR, CAR, dendritic cell, natural killer cell, tumor antigen

## Abstract

Immunotherapy has become a cornerstone in the treatment of cancer and changed the way clinicians and researchers approach tumor vulnerabilities. Durable responses are commonly observed with immune checkpoint inhibitors in highly immunogenic tumors, while the infusion of T cells genetically engineered to express chimeric antigen receptors (CARs) has shown impressive efficacy in certain types of blood cancer. Nevertheless, harnessing our own immunity has not proved successful for most breast cancer patients. In the era of genomic medicine, cellular immunotherapies may provide a more personalized and dynamic tool against tumors displaying heterogeneous mutational landscapes and antigenic pools. This approach encompasses multiple strategies including the adoptive transfer of tumor-infiltrating lymphocytes, dendritic cells, natural killer cells, and engineered immune components such as CAR constructs and engineered T cell receptors. Although far from permeating the clinical setting, technical advances have been overwhelming in recent years, with continuous improvement in traditional challenges such as toxicity, adoptive cell persistence, and intratumoral trafficking. Also, there is an avid search for neoantigens that can be targeted by these strategies, either alone or in combination. In this work, we aim to provide a clinically-oriented overview of preclinical and clinical data regarding the use of cellular immunotherapies in breast cancer.

## Introduction

Breast cancer (BC) is a leading cause of death worldwide and remains mostly incurable in advanced stages (1). Tumor initiation and progression is continuously controlled by innate and adaptive immune cells, which falter as cancer cells undergo mesenchymal dedifferentiation and/or evolve different mechanisms of tumor escape (2). In general, BC is not regarded as an inflamed tumor, triple negative BC (TNBC) and HER2^+^ tumors being more immunogenic than the most common luminal A-like subtype (3). Immunotherapeutic strategies against BC have traditionally been based on “passive immunotherapy” such as the HER2 blocking antibody trastuzumab. Encouraged by the success of immune checkpoint inhibitors (ICIs) in melanoma and lung cancer, numerous trials have tested the use of this “active immunotherapy” in BC with overall disappointing results (4). In the metastatic setting, the most significant achievement was observed in the IMpassion130 phase III trial, which demonstrated an increase in progression-free survival in TNBC patients receiving atezolizumab plus nab-paclitaxel compared to nab-paclitaxel alone (7.2 vs 5.5 months) (5). This humble benefit did not lead to a better overall survival and was not recapitulated when using paclitaxel as concomitant chemotherapy nor consistently associated to any predictive biomarker other than PD-L1 (6). Findings seem to be more clinically meaningful in the neoadjuvant setting, in which an increased pathological complete response rate has been reported in patients receiving atezolizumab (58 vs 41% for total population, 69 vs 49% in PD-L1 positive tumors) (7). This body of evidence underscores the need of a better understanding of the tumor-immune interaction, escape mechanisms, and the role of the microenvironment when a high tumor burden exists. Globally, the use of ICIs in BC would at best provide a nonspecific approach, guided by poorly understood biomarkers, to harnessing a debilitated immune system against a cold tumor. Instead, the development of omic-scale repositories and high-throughput technologies enable us to decode the genomic traits of each unique tumor and calls for the design of more specific and flexible immunotherapies, capable of targeting oncogenic addictions and overcoming temporal and spatial mutational heterogeneities. Thus, the aim of our work is to bridge the complex body of evidence on the different types of adoptive cell therapy (ACT) and the clinicians who everyday care for BC patients.

## T Cell Therapy

### Tumor-Infiltrating Lymphocytes (TIL) Therapy

The adoptive transfer of lymphocytes to treat BC has been attempted in numerous occasions. Allogeneic stem cell transplants in addition to high-dose chemotherapy achieved successful long-term outcomes but arouse significant safety concerns, whereas ACT with autologous circulating lymphocytes conditioned *in vitro* was better tolerated but showed less efficacy (8–11). Tumor-infiltrating lymphocytes (TILs) include a subset of naturally occurring T cells capable of targeting neoantigens encoded by genes harboring nonsynonymus somatic mutations (12, 13). BC, particularly HER2^+^ and luminal-like tumors, have been traditionally considered as poorly immunogenic, with low numbers of TILs and a limited burden of neoantigens (3, 14, 15). However, a robust correlation exists between increased stromal TILs and a better prognosis in TNBC (16–19).

**Figure 1:**
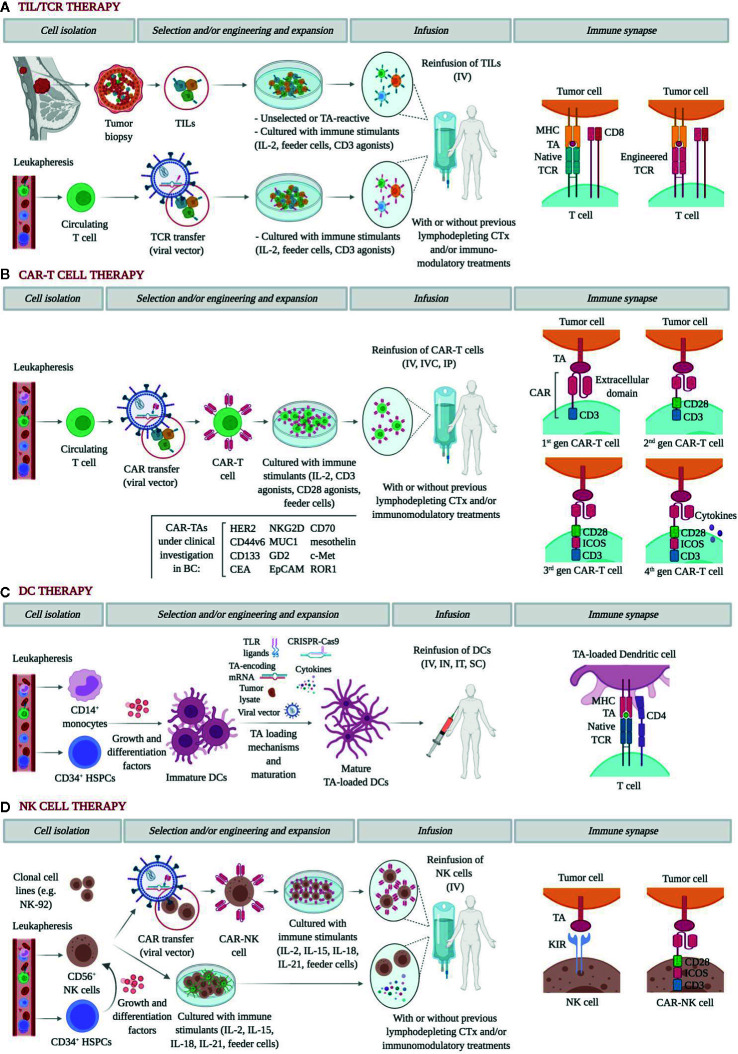
Graphical representation of the main approaches of adoptive cell therapy in breast cancer. **(A)** In general, TILs are enzymatically isolated, activated with high-dose IL-2, and eventually expanded for therapeutic use. More recently, they can also be screened for a high avidity for TAs. TCR transfer, usually using viral vectors on circulating T cells, endows T cells with TCRs with high affinity for TAs. Further, to help condition the body for the T cell transplant, patients often receive a non-myeloablative lymphodepleting chemotherapy regimen before IV infusion, which facilitates the access to growth-promoting cytokines and removes suppressor cells. The role of concomitant immunomodulatory therapies is yet to be elucidated. In both approaches, recognition of cognate TAs is MHC-restricted. **(B)** CAR engineering of circulating T cells has been progressively refined. First-generation CARs include only a CD3ζ chain as intracellular signaling domain; second-generation CARs add a single co-stimulatory domain, such as CD28, 4-1BB (CD137), CD27, or OX40; third-generation CARs add two or three co-stimulatory domains; fourth-generation CARs, also known as TRUCKs (T cells redirected for antigen‐unrestricted cytokine‐initiated killing) are further armored with potent antitumor cytokines and co-stimulatory ligands. CARs target a wide range of surface TAs in an MHC-independent manner, and multiple trials are currently testing the feasibility and efficacy of different administration routes. **(C)** DCs can be generated from PBMNC and HSPCs and become mature after being pulsed using a growing set of TA loading mechanisms. In trials, DCs are infused IV but also as IT or IN vaccines. **(D)** NK cells for ACT can be obtained from clonal cell lines, primary NK cells, or HSPCs. Whether they undergo CAR engineering or remain unmodified, NK cells ligate cognate TAs in an MHC-independent manner. After co-culture with immune stimulants and feeder cells, NK cells are infused IV with or without prior lymphodepleting chemotherapy and/or immunomodulatory treatments. TILs, tumor-infiltrating lymphocyte; IL, interleukin; TA, tumor antigen; TCR, T-cell receptor; MHC, major histocompatibility complex; CAR, chimeric antigen receptor; DC, dendritic cell; PBMC, peripheral blood mononuclear cell; HSPC, hemopoietic pluripotent stem cell; NK, natural killer. IV, intravenous; IVC, intraventricular; IP, intraperitoneal; IN, intranodal; IT, intratumoral; SC, subcutaneous. Figure created with *BioRender.com*.

Adoptive transfer of autologous TILs was first described as a treatment modality by Rosenberg and colleagues in 1987 (**Figure 1A**) (20). Substantial objective responses have been observed in patients with tumors with high mutation rates such as melanoma, lung or bladder cancer (20–22). However, with few exceptions, the infusion of unselected heterogenous TILs appears mostly ineffective in epithelial malignancies (23–27). In order to boost tumor recognition and killing efficacy, TIL therapy has been refined by selecting TILs reactive for tumor antigens (TAs) identified by whole-exome sequencing and RNA sequencing. Zacharakis et al. recently described the case of a 49 year-old woman with ER^+^/HER2^-^ metastatic BC refractory to multiple lines of chemotherapy, who exhibited a complete durable regression after ACT with TA-specific TILs in conjunction with IL-2 and an anti-PD1 agent (28). In this particular case, the genomic analysis of a right breast subcutaneous lesion revealed the presence of 62 nonsynonymous somatic mutations, of which the mutant versions of 4 proteins rendered the highest TIL reactivity. Further, a relevant impact of the concomitant anti-PD1 therapy was unlikely since no expression of PD-L1 was detected in tumor biopsies. A similar approach was used for a pulmonary metastasis of a TNBC patient, where an immunogenic mutation was found among 72 nonsynonymous mutations (29). However, outcome data from this tailored TIL therapy was not reported. Four clinical initiatives have been registered to date in ClinicalTrials.gov and are briefly displayed in **Table 1**. Notably, only two of them incorporate preconditioning with non-myeloablative chemotherapy regimens, and one of them will address the role of an anti-PD1 agent as concurrent medication. In sum, the transfer of selected autologous TILs primed against multiple MHC-restricted TAs may provide a safe and personalized option for patients with advanced BC.

**Table 1:** Clinical trials of ACT in breast cancer.

Antigen	Coadjuvants	Phase	Stage	Phenotype	Route	Precondition	NCT	Status
**TIL therapy**								
Unselected TAs	None	I	IV	TN	IV	Yes	NCT04111510	Recruiting
Unselected TAs	None	I	IV	Mixed	IV	No	NCT01462903	Unknown
Unselected TAs	Anti-PD1	II	IV	Mixed	IV	Yes	NCT01174121	Recruiting
Unselected TAs	Trastuzumab	I	IV	HER2+	IV	No	NCT00301730	Completed
**TCR therapy**								
Neoepitopes	None	II	IV	Mixed	IV	Yes	NCT04102436	Recruiting
Neoepitopes	Anti-PD1	I	IV	HR+	IV	No	NCT03970382	Recruiting
Neoepitopes	Anti-PD1	II	IV	Mixed	IV	Yes	NCT03412877	Recruiting
NYESO-1	None	I	IV	Mixed. HLA-A0201+, NY-ESO-1+	IV	Yes	NCT03159585	Completed
NYESO-1	None	I	IV	Mixed. HLA-A0201+, NY-ESO-1+	IV	Yes	NCT02457650	Unknown
MAGE-A3	None	I/II	IV	HLA-DP0401/02+, MAGE-A3+	IV	Yes	NCT02111850	Active, not recruiting
NYESO-1	None	II	IV	HLA-A2+, NYESO-1+	IV	Yes	NCT01967823	Completed
**CAR-T cell therapy**								
HER2	None	I	IV	HER2+	IV	Yes	NCT04511871	Recruiting
HER2, GD2, CD44v6	None	I/II	III, IV	GD2, CD44v6, HER2+	IV	No	NCT04430595	Recruiting
CD44v6	None	I/II	NR	CD44v6	IV	No	NCT04427449	Recruiting
CEA	None	I/II	IV	CEA+	IV	No	NCT04348643	Recruiting
NKG2D	None	I	IV	TN	IV	No	NCT04107142	Not yet recruiting
MUC1	None	I	IV	TN. MUC1+	IV	Yes	NCT04025216	Recruiting
MUC1	None	I	IV	Mixed	IV	No	NCT04020575	Recruiting
HER2	CAdVEC oncolytic virus	I	Unresectable, IV	HER2+	IV	No	NCT03740256	Not yet recruiting
HER2	None	I	IV (brain, leptomeningeal)	HER2+	IVC	No	NCT03696030	Recruiting
CEA	None	I	IV (carcinomatosis, malignant ascites)	CEA +	IP	No	NCT03682744	Active, not recruiting
GD2	None	I	IV	Mixed	IV	Yes	NCT03635632	Recruiting
EpCAM	None	I	Unresectable, IV	EpCAM+	IV	No	NCT02915445	Recruiting
CEA	Low dose IL-2	I	IV (liver)	CEA+	Hepatic artery	No	NCT02850536	Active, not recruiting
CD70	None	I/II	Unresectable, IV	CD70+	IV	Yes	NCT02830724	Recruiting
Mesothelin	None	I	IV	HER2-. Mesothelin+	IV	Yes	NCT02792114	Recruiting
ROR1	None	I	IV	TN. ROR1+	IV	Yes	NCT02706392	Recruiting
CD133	None	I/II	IV	CD133+	IV	No	NCT02541370	Completed
CEA	Low dose IL-2	I	IV (liver)	CEA+	IV	No.	NCT02416466	Completed
Mesothelin	Anti-PD1	I/II	IV (pleural)	Mesothelin+	Pleural	Yes	NCT02414269	Recruiting
cMet	None	I	IV	TN. cMet+	IT	No	NCT01837602	Completed
**DC therapy**								
HER2/HER3	Anti-PD1, IFNa2b	II	IV	TN, HER2+	SC	No	NCT04348747	Not yet recruiting
Neoepitopes	None	1	II, III	TN	NR	No	NCT04105582	Recruiting
NR	None	I	IV	Mixed	IT	No	NCT03638765	Not yet recruiting
HER2	None	II	I-III, IV in CR	HER2+	IN	No	NCT03630809	Recruiting
NR	None	I/II	IIA, III, IV	Mixed	NR	No	NCT03450044	Completed
HER2	None	I	II, III	HER2+	IN	No	NCT03387553	Recruiting
GFBP2, HER2, IGF1R	None	II	I-III	HER2+	IN	No	NCT03384914	Recruiting
NR	CIK, anti-PD1	I/II	IV	Mixed	IV	No	NCT02886897	Unknown
NR	CIK	II	IV	Mixed	NR	No	NCT02491697	Active, not recruiting
TBVA	None	I	IV	Mixed	SC	No	NCT02479230	Completed
MUC-1	None	I	IV	Mixed	NR	No	NCT02140996	Unknown
HER2	None	I	III (N2)	HER2+	IN	No	NCT02063724	Active, not recruiting
HER2	None	I/II	DCIS	HER2+	IT, IN	No	NCT02061332	Completed
HER2	None	I	I-III	HER2+	IN	No	NCT02061423	Active, not recruiting
Cyclin B1/WT-1/CEF	None	I/II	II-III	TN, ER+	IN, SC	No	NCT02018458	Completed
HER2	None	I	IV	HER2+	SC	No	NCT01730118	Completed
HER2	None	II	II-III	TN, ER+	NR	No	NCT01431196	Completed
WT1	None	I/II	III (N2), IV	TN	SC	No	NCT01291420	Unknown
p53	None	I/II	IV	p53+	SC	No	NCT01042535	Completed
Survivin, hTERT, p53	None	I	IV	Mixed	SC	No	NCT00978913	Completed
OFP/iLRP	None	I/II	IV	Mixed	SC	No	NCT00879489	Unknown
NR	None	II	II-III	TN, ER+	IT, IN	No	NCT00499083	Completed
HER2	None	I	IV	HER2+	SC	No	NCT00197522	Completed
HER2	None	I	Local relapse, IV	HER2	NR	No	NCT00162929	Completed
HER2	None	I	DCIS	HER2+	IN	No	NCT00107211	Completed
p53	None	I/II	III	p53+	SC	No	NCT00082641	Completed
CEA	None	I	IV	Mixed	IV	No	NCT00004604	Completed
**NK cell therapy**								
HER2	None	I/II	IV	HER2+	IV	Yes	NCT04319757	Recruiting
NR	Anti-PD1/PD-L1	I	IV	Mixed	IV	Yes	NCT03841110	Recruiting
NR	None	I/II	All	All	IV	No	NCT03634501	Recruiting
NR	Trastuzumab	I	IV	HER2+	IV	No	NCT03319459	Active, not recruiting
MUC1	None	I/II	IV	TN, MUC1+	IV	No	NCT02839954	Unknown
HER2	Trastuzumab	I/II	IV	HER2+	IV	No	NCT02030561	Unknown
NR	None	II	IV	Mixed	IV	Yes	NCT01105650	Completed

TA, tumor antigen; PD1, programmed death receptor 1; PD-L1, programmed death receptor ligand 1; CIK, cytokine-induced killer cell; TIL, tumor infiltrating lymphocyte; TCR, T-cell receptor; DC, dendritic cell; NK, natural killer; TN, triple negative; HLA, human leukocyte antigen; HR, hormone receptor; IV, intravenous; IVC, intraventricular; IP, intraperitoneal; IN, intranodal; IT, intratumoral; SC, subcutaneous.

### Engineered T Cell Receptor (TCR) and Chimeric Antigen Receptor (CAR) Therapy

Gene transfer-based strategies have been developed to overcome the main challenges of TIL therapy, including the low yield of TIL expansion, the low affinity of human TCRs for TAs, and the immune tolerance elicited by the downregulation of MHC molecules and TAs (30). Both TCR and chimeric antigen receptor (CAR) gene transfer endow polyclonal T cells with reactivities that are not naturally present against TAs of choice and thus provide an adaptable and highly subtle tool for personalized medicine (**Figures 1A, B**) (31).

The majority of engineered αβTCRs recognize epitopes presented by MHC molecules, thereby narrowing down the group of potential targets to those which are MHC-restricted, and exhibit an increased specificity recognition and affinity for tumor cells (**Figure 1A**) (32, 33). Mounting clinical evidence on several tumor types along with preclinical data on BC underscores the rationale for TCR use in BC patients (34–37). Of note, in both hormone-dependent and independent BC cell lines and in xenograft mice, Li et al. reported a notable enhancement of anti-tumor cytotoxicity by CD8^+^ T cells transduced with an MHC-A2-restricted placenta-specific 1 (PLAC1)-TCR molecule (38). However, to the best of our knowledge, evidence on humans is still lacking, with many ongoing clinical trials testing intravenous infusions of TCR-engineered T cells against TAs such as HER2, NYESO-1, and MAGE-A3 (**Table 1**). Interestingly, some of them will assess the value of adding anti-PD1 therapy to enhance immune reconstitution after lymphodepleting chemotherapy and cytotoxicity.

In order to bypass the limitations of MHC restriction of conventional αβTCRs, intensive research has focused on the development of CARs and, more recently, on the γδT cell compartment. γδT cells exhibit potent anti-tumor responses by bridging innate and adaptive immunities, since they incorporate both γδTCRs and killer cell immunoglobulin-like receptors (KIRs) (39, 40). Also, γδT cell ligand recognition requires the expression of accessory costimulatory molecules, which may prevent harmful self-reactivity. Infiltration by γδT cells has been associated with improved outcomes in a small cohort of TNBC patients (41). Consistently, ACT of γδT cells together with trastuzumab improved control of tumor growth as compared to trastuzumab alone in a mouse model of HER2^+^ BC (42). However, the function of the γδT cells may be extremely pleiotropic. In this regard, Peng et al. described a BC-infiltrating γδT cell subset with strong immunosuppressive effects on T cells and DCs regulated *via* the Toll-like receptor 8, thus suggesting that its depletion or reversal could enhance anti-tumor responses (43). ACT with unmodified or engineered γδT cells emerges as an appealing prospect for BC immunotherapy, but further functional characterization and data on clinical interventions are still required (44).

On the other hand, CAR-T cells are T cells engineered to express an artificial receptor with a modular design consisting of an extracellular ligand-binding domain, usually a single-chain antibody, a hinge, a transmembrane domain, and a cytoplasmic signaling domain, with increasing complexity and functionality across the four generations of CAR constructs (**Figure 1B**) (45–47). Compared to TILs, CAR-T cells are not as affected by the hurdles of isolation, expansion, and persistence limitation of natural tumor-specific T cells. Moreover, CAR recognition occurs in an MHC-independent manner, which helps overcome MHC downregulation as a mechanism of tumor escape, and can also recognize carbohydrate and glycolipid antigens (46). Yet, cognate antigens are consequently restricted to surface molecules. Numerous preclinical studies *in vitro* and *in vivo* have evaluated the use of CAR-T cells armed to specifically target TAs in BC, with HER2-CAR constructs attracting the most attention and achieving robust tumor regressions (48–60). To our knowledge, only one phase I trial has been published testing a HER2-CAR in BC patients. In the study by Lum et al., 23 metastatic BC patients independent of their HER status received 8 infusions of anti-CD3/HER2 bispecific antibody-armed T cells. In the evaluable patients at 14.5 weeks, 13 patients experienced clinical benefit, including 2 objective responses (61). Notwithstanding, serious adverse events have been reported following the use of HER2-CARs. The first evidence on the clinical use of HER2-CAR-T cells was a case report of a patient with metastatic HER2^+^ colon cancer in whom the administration of a 3^rd^ generation HER2-CAR was followed by multiple cardiac arrests, respiratory distress, and multiorgan damage (62). This harm was attributed to an inflammatory cytokine release elicited by the immune-mediated recognition of HER2 in normal epithelial tissues, which is referred to as “on-target, off-tumor” toxicity.

Besides HER2, the single injection of accessible lesions with CAR-T cells targeting c-Met, a cell-surface protein tyrosine kinase aberrantly expressed in BC, in a group of 6 patients with metastatic BC comprised by two ER^+^ tumors and 4 TN tumors, did not render measurable responses but elicited extensive tumor necrosis and loss of c-Met immunoreactivity at the injection site, and also translated into detectable levels of c-Met-CAR-T cell mRNA in peripheral blood (63). Similarly, Specht et al. recently communicated preliminary safety results of a phase I trial targeting ROR-1, a tyrosine kinase protein expressed in TNBC and associated with a worse prognosis (64, 65). Interestingly, patients received a 2^nd^ generation ROR1-CAR engineered with a truncated EGFR molecule to permit the elimination of infused cells in case of toxicity (64). Only 6 patients had been enrolled with no adverse events observed, but further update is expected to support this innovative approach.

A considerable number of trials are testing CAR constructs against multiple TAs in BC (**Table 1**). We expect that these studies also convey relevant information about on-target, off-tumor effects, and the benefits of the different administration routes, preconditioning or concomitant immunomodulatory therapies. In addition, it seems clear that a thorough genomic-scale understanding of molecular vulnerabilities and antigenic shifts will be of paramount importance in the design of CAR-based strategies.

## Dendritic Cell (DC) Therapy

Dendritic cells (DCs) are particularly well-suited for BC immunotherapy due to their ability to sensitize CD8^+^ T cells and also CD4^+^ T cells capable of generating memory T cells and contribute with additional cytotoxicity against tumors (66). DCs have been found infiltrating BC specimens in nearly half of the patients with either early or advanced disease, but are mostly relegated to the periphery, functionally compromised, and show a poor correlation with outcome (67–70).

Autologous DCs may be fused with tumor cells or pulsed with tumor lysates or TAs to activate T cells against tumors (**Figure 1C**) (71–74). Across these strategies, DCs may be either exposed to one particular neoantigen or to the entire repertoire of TAs, including those yet to be identified. In contrast to what was observed in TIL and TCR therapies, DCs can be obtained in large numbers from bone marrow precursors and monocyte-derived DCs from peripheral blood (75). The pioneering study by Brossart et al. evaluated the vaccination with autologous DCs pulsed with HER2 or MUC1-derived peptides in 7 BC patients. Although the clinical outcomes were disappointing, peptide-specific T cell responses could be detected even at 9 months after initiation of vaccinations, and T cell responses against epitopes not used for vaccination were identified as a result of cross priming (76). More encouraging objective responses were achieved by Avigan et al. in a phase I trial testing the vaccination with DCs fused with autologous tumor cells in 16 patients with metastatic BC (77). These included 2 patients attaining a partial response and 6 patients attaining a stable disease, although the anti-tumor effects were not maintained over time. In the neo/adjuvant setting, vaccination with autologous HER2-pulsed DCs achieved a modest rate of pathological complete responses in HER2^+^ BC patients, which yet correlated poorly with immune surrogates in peripheral blood (78). This study, however, demonstrated that intralesional and intranodal routes of administration may not substantially differ in terms of anti-tumor efficacy, thus facilitating vaccination when tumor locations are challenging. Likewise, the trial conducted by Qi et al. in stage II-IIIA ER^-^/PR^-^ BC patients reported a 3-year relapse-free survival of 71% versus 31%, with and without vaccination, respectively (79). Other promising approaches consist of adding cytokine adjuvants, such as IL-2, or targeting both the innate and adaptive immune systems by complementing DCs with cytokine-induced killer cells, although the response to these strategies has so far been humble or confused by the effect of concurrent chemotherapies (80–82). More than 20 trials are registered to date testing DC vaccinations in BC patients of all major pathological and most of them are designed to pulse DCs with TAs of choice (**Table 1**). Although ACT with DCs has not yet materialized in a relevant clinical benefit, we believe that the role of DCs as stimulators of T-cell response and long-term memory, and their safety and ease of manufacture, may justify further development alone or in combination with other T cell therapies.

## Natural Killer (NK) Cell Therapy

Different from the previous approaches, NK cells represent an attractive asset for cancer immunotherapy due to their innate ability to eliminate cancer cells in an MHC-independent and non-TA-restricted manner. The “loss of self” mediated by the downregulation of MHC molecules as a mechanism of tumor escape hinders the recognition of cancer cells by CD8^+^ T cells but unleashes the activity of NK cells, which are regulated by the interplay of activating and inhibitory receptors such as KIRs and natural killer group 2D (NKG2D) (83, 84).

Activated NK cells can be manufactured in large numbers *ex vivo* from primary NK cells, hemopoietic stem cells, and clonal cell lines, of which the NK-92 is approved by the US FDA for use in clinical trials (**Figure 1D**) (85–87). So far, adoptive transfer of autologous NK cells has been tested in a wide range of solid malignancies with poor clinical efficacy, which has been explained by the immunosuppressive state of the host and because the inhibitory receptors on autologous NK cells matched molecules exhibited on the tumor cell surface (87–89). Anecdotally, a report by Tian et al. described a partial response in a patient with progressing metastatic HER2^+^ BC who underwent treatment with trastuzumab-treated NK cells, which was consistent with an increased activation and expansion of NK cells mediated by trastuzumab *in vitro* (90). Allogeneic NK cells, however, have not proved to do much better in BC patients, with only one phase II trial published describing 4 patients with stabilized disease from a total of 6 patients evaluated at 4–6 weeks from infusion and after pre-conditioning with lymphodepleting chemotherapy and total body irradiation (91).

In order to enhance their cytotoxic properties, NK cells are also being modified with the addition of CARs against specific TAs. Compared to CAR-T cells, CAR-NK cells are theoretically less potent due to their lack of clonal expansion, relatively short lifespan, and less cytotoxic cytokines (87). Although CAR-T cells may mediate more incisive and long-term responses, the use of CAR-NK cells would minimize the risk of cytokine release syndrome and tumor-lysis syndrome, thereby increasing overall treatment safety (92). Importantly, CAR-NK therapy is expected to be much less expensive, considering that NK cells can be derived from multiple sources. Encouraging results have been reported in a phase I/IIa trial using cord blood-derived CAR-NK cells targeting CD19 in patients with relapsed or refractory non-Hodgkin’s lymphoma and chronic lymphocytic leukemia, with up to 64% of patients achieving a complete response (93). In BC, tissue factor (TF) was recently described by Hu as a novel and common yet selective molecule on TNBC, whose targeting by TF-CAR NK cells resulted in an increased cytotoxicity against TNBC cell lines and was effective and safe for the treatment of TNBC in an orthotopic mouse model (94). Chen et al. recapitulated these findings when investigating the effect of EGFR-CAR NK cells in TBNC cell lines and in mice pre-inoculated with brain metastases (95). To the best of our knowledge, there is not published data on human trials on BC to date, although several initiatives can be found registered in the Clinical Trials.gov repository including multiple trials evaluating the intravenous infusion of *ex vivo* expanded, autologous NK cells and also the administration of NK cells incorporating HER2- and MUC1-CAR constructs (**Table 1**).

## Concluding Remarks

ACT offers a growing toolkit to overcome antigenic heterogeneity and the broad repertoire of immune escape mechanisms occurring in advanced BC. To fully capitalize these set of highly personalized treatments, we must address both approach-specific and cross-cutting challenges. ACT with autologous TILs may benefit from the standardization of TIL assessment in routine biopsies and the effective expansion of those TILs with the highest anti-tumor reactivity. Gene transfer-based TCR therapies increase antigen specificity but still fail to target those not presented by the MHC, whereas CAR engineering may provide additional versatility but entails elevated costs and significant on-target, off-tumor toxicity. Additionally, although DC and NK cell therapies may have not achieved relevant tumor responses, their better safety profile and reduced costs make them suitable companions for multimodal strategies. The successful transition of the different ACTs to the clinic poses a number of common considerations. The discovery of TAs that can guide ACT against BC is critically linked to its success and relies on comprehensive strategies integrating genomic sequencing, *in silico* prediction, and immunogenicity evaluation. Methodological refinement is also required to improve our ability to isolate immune components and modify them *ex vivo* and *in vivo*, and to enhance cell persistence and intratumor trafficking. Finally, clinical trials testing ACTs will progressively need to be more adaptable, explore the reliability of predictive biomarkers, and generate quality data from small sample sizes. Both puzzling and fascinating, this is the path ahead to materialize ACT and transform the therapeutic landscape of BC patients.

## Author Contributions

JF-A, KG-H, and AO contributed to the conception and scope of the study. JF-A and AO wrote the first draft. JF-A and KG-H composed the figures/tables. All authors contributed to the article and approved the submitted version.

## Conflict of Interest

The authors declare that the research was conducted in the absence of any commercial or financial relationships that could be construed as a potential conflict of interest.
